# Supplementary material for: A Polysaccharide-RBD-Fc-Conjugated COVID-19 Vaccine, SCTV01A, Showed High Immunogenicity and Low Toxicity in Animal Models
Source: Vaccines (Basel). 2023 Feb 23;11(3):526. doi: 10.3390/vaccines11030526 (PMC10052645; doi:10.3390/vaccines11030526)
Supplement: Supplementary file 1 [file vaccines-11-00526-s001.zip › vaccines-2202205-supplementary.pdf]

Supplementary Information

A Polysaccharide-RBD-Fc-Conjugated COVID-19 Vaccine, SCTV01A, Showed High Immunogenicity and Low Toxicity in Animal Models

Chunyun Sun [1,†], Desheng Kong [1,†], Erhong Guo [1], Jun Zhao [1], Jilei Jia [1], Rui Wang [1], Juan Ma [1], Meng Chen [1], Jianbo Lu [1], Chulin Yu [1], Kuokuo Li [1] and Liangzhi Xie [1,2,3,*]

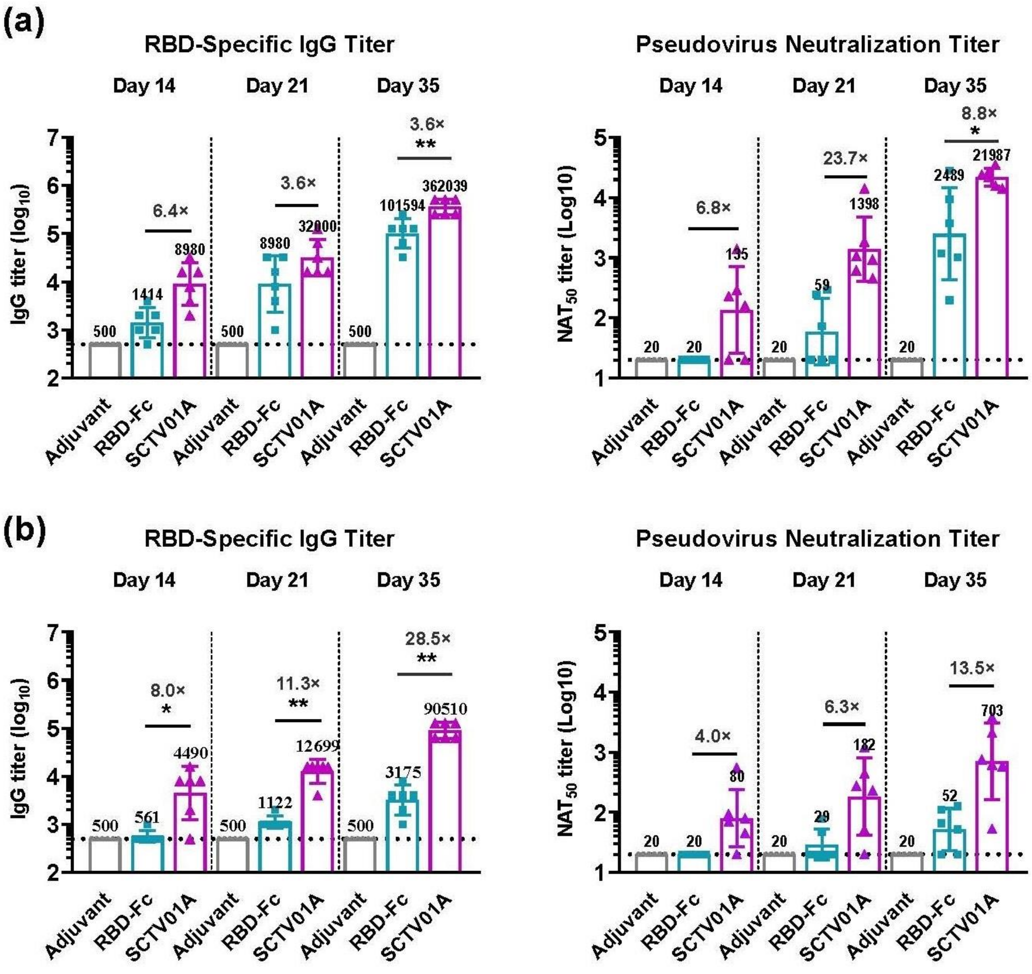

Figure S1. SCTV01A enhances RBD-specific antibody titer and NAT_{50} titer in C57BL/6 mice. C57BL/6 mice (n=6/group) were intramuscularly immunized with 1 μg of SCTV01A or RBD-Fc formulated with SCT-VA02B (**a**) or Alum (**b**). The GMT of RBD-specific antibody titer and NAT_{50} titer against the Wuhan-Hu-1 strain were detected as described above. Statistical

analyses were performed using unpaired two-tailed Welch's tests. *$p \le 0.05$ and **$p \le 0.01$ represent statistical significance.

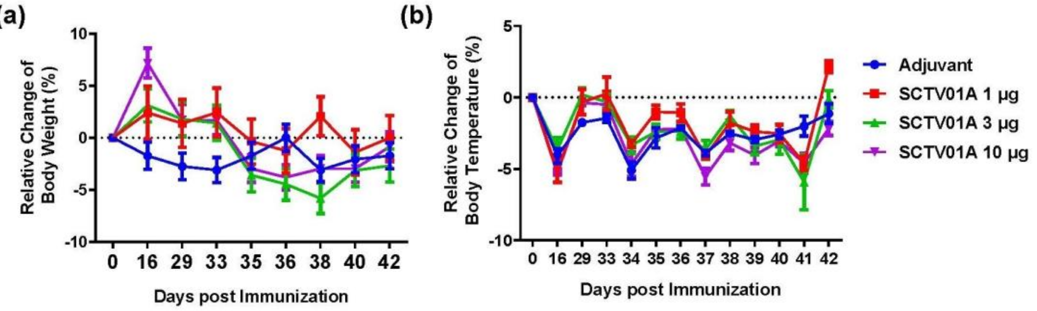

Figure S2. Relative Change of Body weight and body temperature in Rhesus Macaques at different time points after SCTV01A immunization. Rhesus macaques (n=4/group) were immunized with 1 μg, 3 μg, 10 μg of SCTV01A adjuvant with SCT-VA02B at Day 0, Day 16 and Day 29. Same volume of SCT-VA02B was immunized as control. Body weight (**a**) and body temperature (**b**) in rhesus macaques at different time points after SCTV01A immunization. All data are presented as mean ± SD.

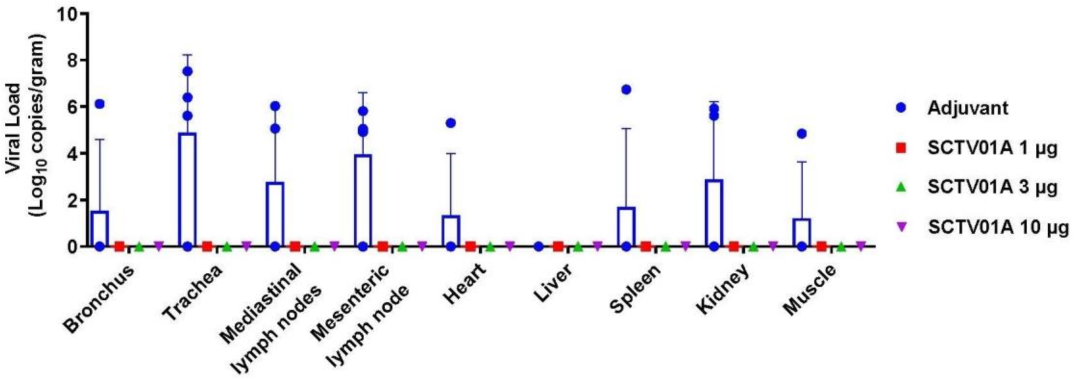

Figure S3. SARS-CoV-2 viral load of different tissues was determined using RT-qPCR at 7 dpi.

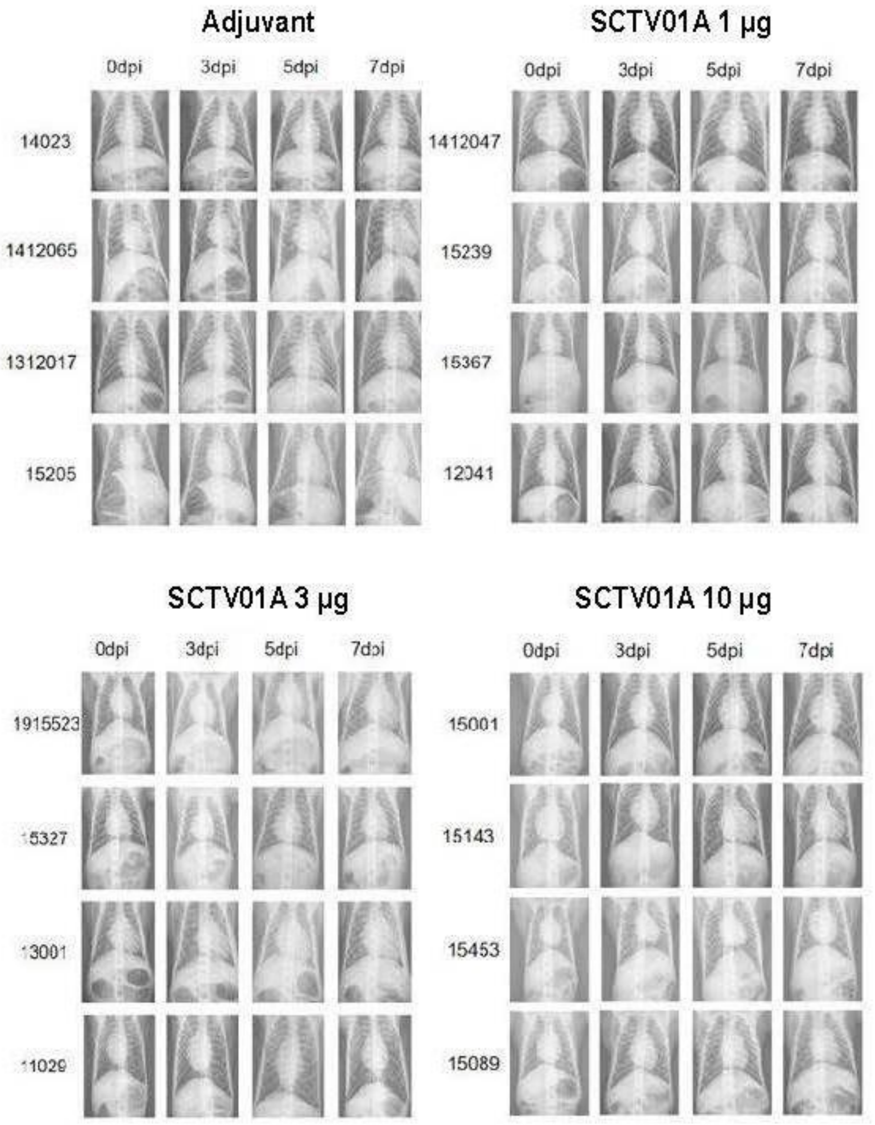

Figure S4. Chest X-ray images in Rhesus Macaques at different time points after SARS-CoV-2 infection. Individual X-ray image of Rhesus Macaques at 0, 3, 5, 7 dpi after SARS-CoV-2 infection.

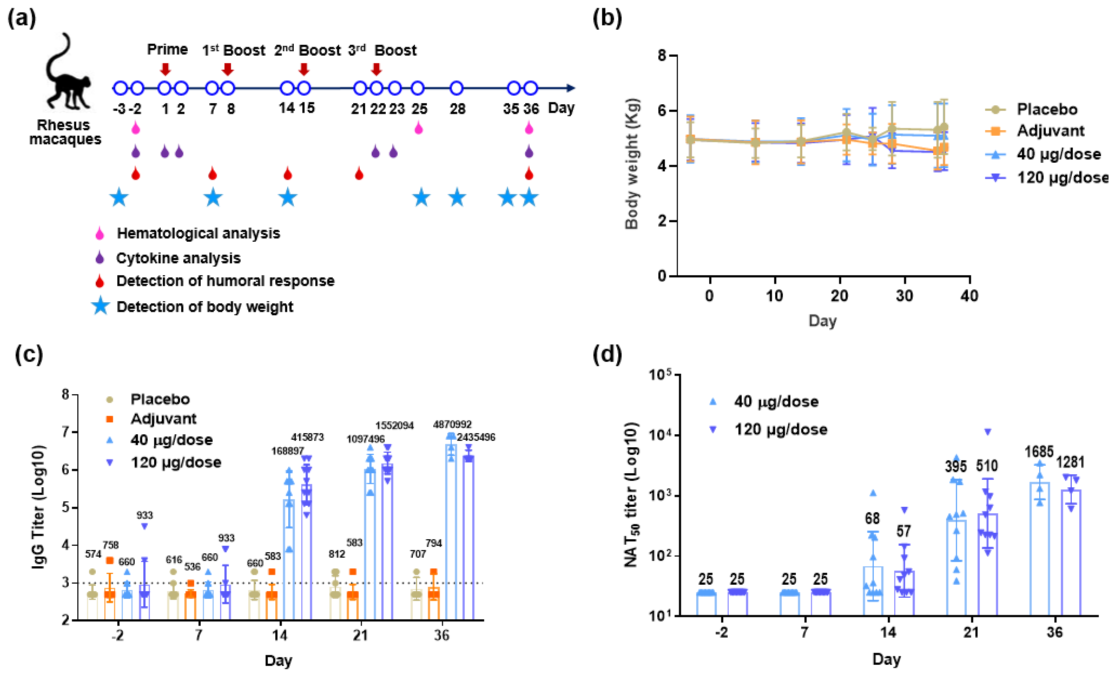

Figure S5. Body weight and immunogenicity were monitored to evaluate the safety of SCTV01A in nonhuman primates. (**a**) Schematic representation of the immunization, blood collection and body weight detection schedule for the safety study in rhesus macaques. During the study, parameters evaluated in the study included the clinical observations, body weight, body temperature, electrocardiogram (ECG), blood pressure, blood oxygen saturation, ophthalmology, clinical pathology (hematology, coagulation, clinical chemistry, urine analysis), lymphocyte subsets ($CD3^+$, $CD4^+$, $CD8^+$, $CD4^+CD8^+$, and $CD20^+$), serum cytokines (IFN-γ, TNF-α, IL-2, IL-4 and IL-6), serum complements (C3, C4), and the specific IgG antibody and the nAb against SARS-CoV-2 recombinant protein. (**b**) Body weight, (**c**) RBD-specific IgG titer and (**d**) PsV NAT_{50} titer were monitored at different time points.

Table S1. Lung pathological grading criteria.

Grade	Pathological Changes
0	Alveolar structure is clear, no inflammation infiltration
1	Mild inflammation, slightly widened alveolar septum and rare mononuclear cells including monocytes and lymphocyte infiltration
2	Obvious inflammation, thickening of alveolar wall, increased inflammatory infiltration of interstitial monocytes
3	Alveolar space is significantly widened, and inflammatory cell infiltration increases
4	Extensive exudation and interval widening, alveolar cavity becomes smaller, interval bleeding is obvious, more cell infiltration in alveolar cavity
>5	A large number of cells infiltrate the alveolar cavity, the alveolar cavity disappears, the interval is fused into a piece, and the alveolar wall is formed with a transparent membrane
